# Understanding missed opportunities for more timely diagnosis of cancer in symptomatic patients after presentation

**DOI:** 10.1038/bjc.2015.47

**Published:** 2015-03-03

**Authors:** G Lyratzopoulos, P Vedsted, H Singh

**Affiliations:** 1 Health Behaviour Research Centre, Department of Epidemiology and Public Health, University College London, 1-19 Torrington Place, London WC1E 6BT, UK; 2 Department of Public Health and Primary Care, Cambridge Centre for Health Services Research, University of Cambridge, Institute of Public Health, Forvie Site, Robinson Way, Cambridge CB2 0SR, UK; 3 Department of Public Health, Research Unit for General Practice, Research Centre for Cancer Diagnosis in Primary Care (CaP), Aarhus University, DK-Bartholins Allé, 8000 Aarhus, Denmark; 4 Houston Veterans Affairs Center for Innovations in Quality, Effectiveness and Safety, Michael E. DeBakey Veterans Affairs Medical Center and the Section of Health Services Research, Department of Medicine, Baylor College of Medicine, Houston TX 77030, US

**Keywords:** neoplasm, diagnosis, missed opportunities, patient safety, general practice, system factors, errors, quality

## Abstract

The diagnosis of cancer is a complex, multi-step process. In this paper, we highlight factors involved in missed opportunities to diagnose cancer more promptly in symptomatic patients and discuss responsible mechanisms and potential strategies to shorten intervals from presentation to diagnosis. Missed opportunities are instances in which *post-hoc* judgement indicates that alternative decisions or actions could have led to more timely diagnosis. They can occur in any of the three phases of the diagnostic process (initial diagnostic assessment; diagnostic test performance and interpretation; and diagnostic follow-up and coordination) and can involve patient, doctor/care team, and health-care system factors, often in combination. In this perspective article, we consider epidemiological ‘signals' suggestive of missed opportunities and draw on evidence from retrospective case reviews of cancer patient cohorts to summarise factors that contribute to missed opportunities. Multi-disciplinary research targeting such factors is important to shorten diagnostic intervals post presentation. Insights from the fields of organisational and cognitive psychology, human factors science and informatics can be extremely valuable in this emerging research agenda. We provide a conceptual foundation for the development of future interventions to minimise the occurrence of missed opportunities in cancer diagnosis, enriching current approaches that chiefly focus on clinical decision support or on widening access to investigations.

The diagnosis of cancer in symptomatic patients requires a complex, multi-step process. Consequently, some patients experience prolonged intervals to diagnosis, which arise from various patient, doctor and health-care system-related factors involved in this process. Prompt diagnosis in symptomatic cancer patients represents a core deliverable of modern health-care systems, and the public (including patients, politicians and the media) considers it a serious priority. However, tensions between societal expectations for prompt cancer diagnosis in all patients and the challenges involved in achieving this aim are being increasingly recognised by patient groups, health-care professionals and policy makers. Research on strategies to minimise prolonged diagnostic intervals after presentation has become a priority. The objectives of this article are to highlight factors involved in missed opportunities for cancer diagnosis among symptomatic patients and discuss potential mechanisms and approaches to accelerating progress towards minimising diagnostic delays post presentation. We provide a conceptual foundation for developing multi-faceted strategies to achieve timely diagnosis in the greatest possible numbers of patients post presentation.

## Retrospective case analyses suggestive of missed diagnostic opportunities

A large body of evidence based on analysis of the clinical details of cohorts of cancer patients suggests that ‘missed opportunities' occur in substantial proportions of patients (Singh *et al*, 2007, 2009a, 2010). A related concept is that of ‘quality deviations' in the diagnosis of cancer (Jensen *et al*, 2014). A recent UK study also documents missed opportunities (Mitchell *et al*, 2013). Missed opportunities are instances in which *post-hoc* judgement indicates that alternative decisions or actions could have led to more timely diagnosis—i.e., something different could have been done or considered under the given circumstances to reach a more prompt diagnosis (Singh, 2014). For example, absence of evaluation for possible gastrointestinal bleeding in a 50-year-old man with new onset iron deficiency anaemia could represent a missed diagnostic opportunity for more timely diagnosis of gastrointestinal cancer. Determining the presence of missed opportunity involves a process of retrospective adjudication typically based on patient record audits, but it helps uncover critical areas for improvements in diagnostic quality.

**Figure 1:**
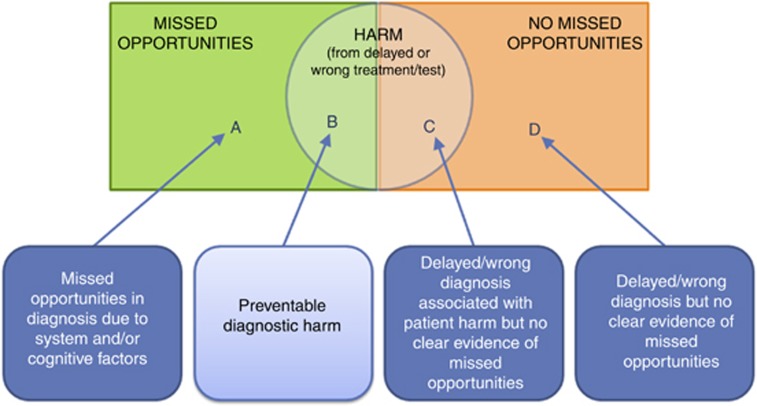
**A model for defining missed diagnostic opportunities.** Adopted from Singh, 2014.

It should be emphasised that not all missed opportunities or delays necessarily result in harm or poor patient outcomes and not all instances of delayed diagnosis are associated with missed opportunities (Figure 1; Singh, 2014). Missed opportunities may relate to any disease; for example, six out of seven patients subsequently diagnosed with chronic obstructive pulmonary disease are reported to have experienced missed opportunities, and very long diagnostic delays are reported among patients with ankylosing spondylitis (Hamilton *et al*, 2011; Jones *et al*, 2014). In this paper nonetheless we focus solely on patients with cancer who have sought medical help for their symptoms and exclude the consideration of potential opportunities for earlier presentation or participation in screening programmes.

## Epidemiological signals of missed diagnostic opportunities

**Figure 2:**
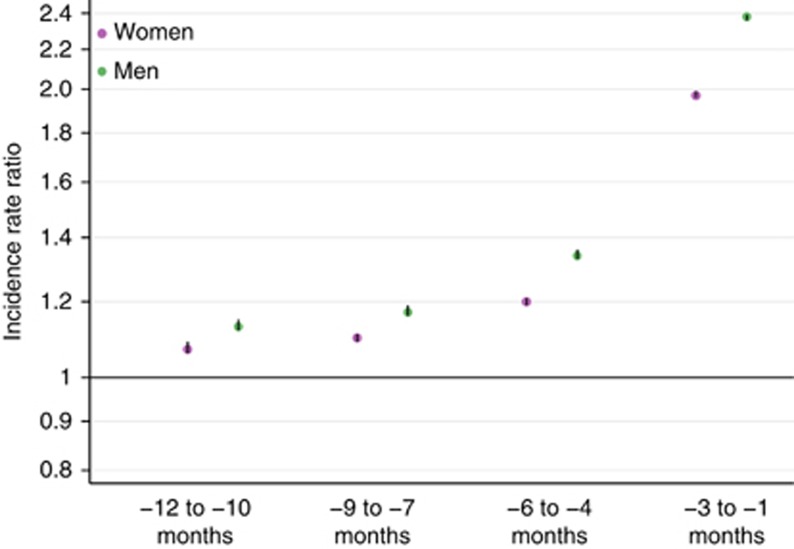
**Epidemiological evidence suggestive of likely missed opportunities.** Incidence rate ratios (IRR) for general practitioner consultations before the diagnosis of cancer compared with age- and sex-matched ‘control' patients (without a diagnosis of cancer). Data from Christensen *et al* (2012); *n* (women)**=**63 362 cancer patients and 633 620 controls; *n* (men)=63 848 cancer patients and 638 480 controls. Note very narrow 95% confidence intervals that exclude parity (i.e., 1.00); and excess risk spanning a 12-month period, including −6 to −4 months.

Retrospective evaluation of individual cases is not the only source of evidence on missed diagnostic opportunities. In England, work conducted by the (former) National Patient Safety Agency has highlighted factors implicated in diagnostic delays in cancer and recommended ‘routine monitoring of delayed diagnosis' (NPSA, 2010). Although thus far no system that allows such routine monitoring exists, epidemiological evidence from England indicates that about one in five patients seen in general practice and subsequently diagnosed with cancer consults with their general practitioners three or more times for relevant symptoms before a specialist referral is made and that instances of multiple consultations are associated with prolonged primary care intervals (Lyratzopoulos *et al*, 2012, 2013a). Similarly, evidence from Denmark indicates that the rate of primary care consultations, diagnostic tests and hospital visits among patients subsequently diagnosed with cancer is substantially higher than that of ‘control' patients (without cancer), over a period of several months before diagnosis (Figure 2) (Christensen *et al*, 2012; Ahrensberg *et al*, 2013; Hansen *et al*, 2015). However, epidemiological studies do not provide direct evidence or specific clinical information about the circumstances surrounding such events, and not all instances of these ‘early'/multiple pre-diagnostic consultations would be associated with missed opportunities. For example, multiple consultations may be unavoidable in the presence of vague symptoms and/or when it is judged reasonable to investigate patients before referral (Lyratzopoulos *et al* 2014). Nonetheless, this type of evidence strongly indicates that missed opportunities may occur in at least some patients with cancer diagnoses and we need to understand more about their origins, both in primary and secondary care and throughout the diagnostic process. Further, epidemiological evidence can help identify cancer sites or socio-demographic characteristics of patients that confer a higher than average risk of delayed diagnosis (Lyratzopoulos *et al* 2012, 2013a), providing insights into potential responsible mechanisms and targets for further research and improvement initiatives.

## Types and origins of missed opportunities in cancer diagnosis

Missed opportunities for diagnosing cancer sooner may occur anywhere in the diagnostic process. On the basis of evidence from retrospective case reviews of cohorts of cancer patients, missed opportunities typically occur in three main phases:

1. **Initial diagnostic assessment** (during the clinical encounter between a patient and a doctor, typically, but not exclusively, a generalist). This phase involves history taking, clinical examination and diagnostic reasoning, potentially also leading to specialist referral, test ordering or expectant (‘safety netting'/‘wait and see') management decisions, or their combination.
2. **Diagnostic test performance and interpretation**. This phase involves the process of performing appropriate diagnostic tests (e.g., blood tests, imaging or endoscopy, often at different times and locations) and their appropriate interpretation and associated actions.
3. **Diagnostic follow-up and coordination**. This phase includes many activities and tasks required to ‘close the loop' on test results and referrals made on initial diagnostic assessment.

Patient, provider and system factors can all contribute to the generation of missed opportunities during one or more of the above phases, and missed opportunities in diagnosis often involve more than one contributory factor (Singh *et al*, 2013a). Complex interactions exist between these factors; for example, both patient and doctor factors could be influenced by system factors (Andersen *et al*, 2014). Understanding the complex interplay between these factors is important for reducing missed opportunities, thus underscoring the importance of using multi-disciplinary approaches in this area, including perspectives from psychology, human factors (the scientific field that focusses on how people interact with products, tools, procedures and processes) and informatics.

The concept of missed diagnostic opportunities builds on previous theoretical models from psychology. For example, the ‘model of pathways to treatment' provides a holistic consideration of the journey from symptom onset to diagnosis and treatment initiation, encompassing four distinct intervals (symptom appraisal, help-seeking, diagnostic and pre-treatment intervals), with the diagnostic interval being of relevance to missed opportunities after presentation as considered in this paper. (Walter *et al*, 2012; Scott *et al*, 2013).

We use the high-level taxonomy of phases described above toillustrate different types of missed opportunities and related contributing factors to inform and motivate further policy initiatives and research.

### Missed opportunities and contributing factors during initial diagnostic assessment

#### Rigid consultation norms

In some countries (including the UK and Denmark) medical consultation norms encourage patients to consult for ‘one problem at a time' (which may even have to be declared in advance, before consultation), while the duration of primary care appointments is typically as short as 10 min (National Health Service Information Centre, 2007; Andersen *et al*, 2014; McCartney, 2014). Further, notable proportions of the public in countries with publicly funded health-care systems worry about consulting for symptoms that may ‘waste the doctor's time' (Forbes *et al*, 2013). Beyond increasing the risk of delayed presentation and help-seeking, such attitudes might also decrease patient resolve to use up consultation time for communicating the full breadth and complexity of their symptoms, thereby increasing the risk of missed opportunities (Andersen *et al*, 2011).

#### Inadequate history taking and examination

For several reasons, the full spectrum, nature and duration of symptoms may not be elicited during a primary care encounter. Retrospective medical record reviews of patients diagnosed with cancer indicate that insufficient symptom elicitation or recording and ineffective doctor-patient communication may account for many instances of missed opportunities (Singh *et al*, 2009a; Jensen *et al*, 2014). Time pressures, either real or perceived, may impede doctors to obtain a thorough history or elicit clinical signs when present (Andersen *et al*, 2014; McCartney, 2014). Other factors described below might also contribute.

#### Language barriers

An increasing number of cancer patients in Europe and North America have limited proficiency in the first language of their resident country. In such circumstances, lack of interpretative support may impede effective patient–doctor communication, with some epidemiological evidence suggesting that suspecting the diagnosis of cancer is less prompt (i.e., requiring a greater number of pre-referral consultations) in older ethnic minority patients with symptoms (Lyratzopoulos *et al*, 2012).

#### Cognitive factors impeding optimal initial clinical assessment and reasoning

Firmly suspecting the diagnosis of cancer during a single clinical encounter is difficult, as symptoms and signs are rarely pathognomonic and may also be seen early in their development (Jones *et al*, 2007; Hamilton 2009; Jones *et al* 2009). Furthermore, as public awareness campaigns, by their very nature, encourage larger proportions of persons with symptoms to consult, the already low positive predictive value of symptomatic presentations in primary care for cancer may decrease further. A range of factors may, however, make diagnostic reasoning even more challenging. These include the following:

- Cognitive biases: several types of such biases exist, including anchoring bias (focusing exclusively on a single item of information), availability bias (over-reliance on already known or easily available information) and ‘commitment to a steer' (i.e., initial diagnostic impressions), which can impede diagnostic reasoning (Kostopoulou *et al*, 2012; Croskerry 2013). In a study of diagnostic errors in UK primary care, biases at the initial framing of the problem were related to errors at the end of the diagnostic process (Balla *et al*, 2012). In addition to operating during the clinical encounter, these biases could also lead to the misinterpretation of diagnostic test results (Singh *et al*, 2012a).
- Co-morbidity: consideration of a cancer diagnosis is particularly challenging in the presence of other known non-cancer co-morbid conditions. Many older patients (the age group at higher risk for cancer) are multi-morbid (Barnett *et al*, 2012). In these patients, symptoms compatible with the known cause of chronic morbidity could be easily thought to reflect the pre-existing disease rather than a new problem (Mitchell *et al*, 2013).
- Unfamiliarity with cancer presentations: patients with a new diagnosis of cancer are infrequent in general practice. For example, in the UK a full-time general practitioner on average might encounter only between 5 and 10 new cases in a year, amid thousands of patients with other conditions. Beyond unfamiliarity, ‘epidemiological optimism' bias can make prompt suspicion of the diagnosis of cancer even harder in low-risk patient groups even when they complain of symptoms that may be due to cancer. Such groups include young persons and certain socio-demographic groups within specific cancers (e.g., women who present with visible haematuria; Lyratzopoulos *et al*, 2012, 2013b; Nicholson *et al*, 2014).

#### Access and system capacity constraints

These are often expressed as long waiting times. An indirect consequence of prolonged waiting times is that they *de facto* increase the disease severity threshold for referral decisions—i.e., capacity constraints influence doctor-decision making. This may be a particular challenge for publicly funded systems where demand management functions are implicitly delegated to primary care services (Vedsted and Olesen 2011; Brown *et al*, 2014). Geographical barriers, such as distance to diagnostic centres, may also be relevant, although evidence on such associations is needed.

#### Referral norms

The positive predictive value of signs and symptoms for cancer is low; only a few have values >5% in patients presenting in general practice (Shapley *et al*, 2010). Consequently, most patients investigated for suspected cancer will not have the disease; for example, in the UK among patients who are referred to specialists for suspected cancer, about 90% will be found not to have cancer (Meechan *et al*, 2012). Likely peer pressure by specialists or hospital managers may increase reluctance by primary care doctors to refer patients if there is intolerance of low ‘diagnostic hit rates', increasing the risk of missed opportunities, although evidence to further establish the role of such dynamics would be desirable.

### Missed opportunities and contributing factors during diagnostic test performance and interpretation

#### Patient non-adherence with recommended tests and lack of system resilience towards such (‘no show') events

Patients may not adhere to prescribed investigation plans, either by not attending recommended investigations or by not preparing for them. For example, patient non-adherence with suggested colonoscopy investigation can lead to post-referral diagnostic delays in colorectal cancer (Singh *et al*, 2012b). In Denmark, among all missed opportunities, 16% were attributable to patients not showing up (Jensen *et al*, 2014). Different factors may be implicated in ‘no show' events. Evidence from patients not adhering to screening colonoscopy appointments implicates emotional barriers (such as fear of an adverse diagnosis, or fear of procedure-related pain or complications) and logistical or communication barriers (Denberg *et al*, 2005), and similar factors may be applicable to diagnostic colonoscopies. When ‘no show' events do occur, communication with the patient and a review of the patient's management should be automatically triggered to reschedule investigations or initiate alternative management.

#### Diagnostic testing process complexity

The diagnosis of cancer typically requires a sequence (‘chain') of tests and procedures (e.g., blood tests, imaging, endoscopy leading to tissue sampling and pathology reporting). These tests are by necessity often carried out at different locations and times, whereas generalist and specialist doctors often work in different settings, adding levels of complexity to the diagnostic process. This high degree of complexity multiplies the risk of delays and/or erroneous decision making at different steps in the process by a factor proportionate to the number of distinct tests required. Further, the distribution of the diagnostic process (in space and time, and between primary care and specialist care, often including different departments and locations) increases both time lags between different steps in the process and risks of miscommunication (Jensen *et al*, 2014), untimely communication or lack of follow-up of important test results. This has, for example, in Denmark, led to the formation of diagnostic units that enable the conduct of multiple required tests and specialist assessments ‘within 1 day/under one roof', and similar services are currently being developed in the UK. Such strategies will need to be evaluated for their effectiveness. Earlier work by the National Patient Safety Agency has recommended that the health service needs to ‘identify, review and disseminate current good practice in the process of ordering, managing and tracking tests and test results' (NPSA, 2010).

#### Inadequacies in the investigation strategy (initially negative tests in the presence of ongoing symptoms or diagnostic suspicion)

This may occur when the suspicion of cancer is correctly raised but decisions on planned investigations are sub-optimal or inadequate (Jensen *et al*, 2014). This scenario may be more likely for cancers sharing many common symptoms (e.g., cancers of pelvic/abdominal organs). For example, a patient with abdominal symptoms is investigated with a colonoscopy that is negative, and this finding is initially interpreted as bringing ‘diagnostic closure', but the patient has persistent symptoms and is subsequently found to have cancer of another abdominal organ (pancreas, liver or ovary). Another example can be provided by false-negative chest X-ray findings in patients with suspected lung cancer (Bjerager *et al*, 2006; Stapley *et al*, 2006). Such circumstances can clearly prolong diagnostic intervals by providing (temporary) false reassurance (Singh *et al*, 2012a). A much more challenging situation occurs when correct tests have been carried out but the results are falsely interpreted as negative, without adequate fail-safe or back-up re-assessment mechanisms being present (Singh *et al*, 2012a; Middleton *et al*, 2014).

### Missed opportunities and contributing factors during follow-up and coordination

#### Patient factors

If appropriately empowered, patients' active role in the diagnostic process can minimise the risks of missed opportunities. How this potential can be harnessed should be a priority for future research (McDonald *et al*, 2013). The National Patient Safety Agency report on diagnostic delays in cancer recommended that the health service ‘develop methods for empowering patients on a cancer diagnostic pathway' (NPSA, 2010). However, currently:

- Many patients do not feel empowered to seek out the results of tests performed on them, or do not know how to do so. Patients may also be ‘reassured' by lack of follow-up by doctors/the health-care system, interpreting lack of communication to mean that ‘all is normal' in instances when this is not the case. This emphasises the importance of passive (‘open door') or active (fixed interval, e.g., 3-week clinical review) follow-up as part of safety-netting strategies (Almond *et al*, 2009).
- Patients also might not be willing to re-consult or seek a second medical opinion despite doubting the certainty of their diagnosis and persistent or worsening symptoms (Birt *et al*, 2014; McDonald *et al*, 2013).
- Another common occurrence is patients not returning for ‘fail-safe/safety-netting' visits planned as part of expectant management strategies, and/or when they experience persistent, worsening or new symptoms (Singh *et al*, 2012a; Mitchell *et al*, 2013). Factors similar to those involved in non-adherence with diagnostic investigations may be implicated (see ‘Missed opportunities and contributing factors during diagnostic test performance and interpretation', above). Robust mechanisms for identification of such occurrences and contact with patients are required.

#### Over-reliance on patients to ‘call back'

Doctors often believe that patients will call if they do not feel better or new symptoms develop, and often assume that the diagnosis they had recently given had been correct if they do not hear otherwise (Singh and Sittig, 2014). Ensuring timely patient follow-up could also help prevent missed opportunities that relate to coordination failures between different clinics, hospital departments and general practices (Mitchell *et al*, 2013). Proactive follow-up systems and protocols that leverage information technology might be needed to minimise the risk of such missed opportunities ‘at the last hurdle' (Murphy *et al*, 2014).

#### Lack of appreciation or follow-up of abnormal test results

Increasingly recognised in ‘electronic health record-enabled' health-care systems are instances of failure to recognise and act on abnormal tests related to cancer (Murphy *et al*, 2014). Many reasons could contribute to such occurrences—for example, physician ‘alert fatigue' or ambiguities about the health-care professional who is ‘in charge' of the patient and responsible for follow-up (Singh *et al*, 2009b). Informational continuity and clarity of accountability for the patient as they progress through the diagnostic pathway poses remarkable challenges (Press, 2014). Because modern health care is delivered by teams rather than by individuals, factors relating to team dynamics and ‘distributed cognition' could also be relevant; these include low staff morale, poor communication between team members and limited ‘situational awareness' of diagnostic safety (Singh *et al*, 2012a).

## Measurement, learning and improvement

As is the case for safety interventions in general, identification of instances where missed opportunities have occurred is a pre-requisite for motivating and guiding organisational learning and improvement efforts and service redesign (Donaldson *et al*, 2014). Although we acknowledge that not all missed opportunities or delays would directly result in poorer patient outcomes (see also Figure 1), we believe that all preventable diagnostic delays should be avoided. Additional reasons to do so include strong patient (and carer) preferences for prompt diagnosis of cancer and substantial burden of patient complaints and medico-legal claims associated with diagnostic delays/missed diagnostic opportunities (Gandhi *et al*, 2006; Schiff *et al*, 2013).

Measurement of missed opportunities relies on operational definitions that may vary between settings and systems. Ideally, measures with optimal construct validity should be developed (Singh and Sittig, 2015). However, this is difficult to achieve given the current state of the evidence and scientific knowledge related to diagnostic safety. We therefore suggest that resources should be prioritised for developing and using ‘surrogate markers' of missed opportunities, which could ‘trigger' clinical audit activity and case reviews to verify the presence and nature of missed opportunities and likely contributory factors. Different possible surrogate markers (or triggers) have been proposed in the literature, including unusual patterns of multiple consultations (‘return visits'), emergency presentations (Elliss-Brookes *et al*, 2012; Lyratzopoulos *et al*, 2012; Mitchell *et al*, 2013; Singh *et al*, 2013a; Jensen *et al*, 2014) or symptoms or abnormal test findings suggesting the need for diagnostic evaluation for cancer (Murphy *et al*, 2014). Another approach is doctor-led retrospective reviews of all or randomly selected cases of cancer diagnosed in a practice or health centre during defined periods (Baughan *et al*, 2009; Rubin *et al*, 2011; Mitchell *et al*, 2013). Patient record audits offer several advantages, including detailed information and longitudinal data about symptoms, investigations and diagnosis evolution, but their limitations include the potential for hindsight bias, missing documentation and review time (Zwaan *et al*, 2010). Although no criterion or method to define missed opportunities is 100% sensitive and specific, the use of markers/triggers can motivate and support quality improvement activities and professional and organisational learning.

We summarise common factors involved in missed opportunities and list a range of possible actions in Table 1, acknowledging that evidence on the effectiveness of cognitive and system interventions is still emerging and there is a need for further research and evaluation in relevant areas (Graber *et al*, 2012; Singh *et al*, 2012c).

## Implications for research and policy. enriching the spectrum of post-presentation interventions to promote early diagnosis

Recently, an Institute of Medicine (IOM) committee was tasked with evaluating diagnostic error as a patient safety issue (IOM, 2014). As this new report will be a continuation of the IOM's Health Care Quality Initiative report ‘To Err is Human: Building a Safer Health System *and* Crossing the Quality Chasm: A New Health System for the 21st Century', the importance of early diagnosis and of improving diagnostic safety for both researchers and policy-makers is likely to increase substantially in the coming years.

Several policies aimed at expediting the diagnosis of cancer after presentation to general practitioners chiefly emphasise either ‘knowledge mobilisation' interventions (e.g., reminding primary care physicians of the possibility of cancer diagnosis during consultations), or enabling of greater access to endoscopy or imaging investigations (Department of Health, 2012; Hamilton *et al*, 2013). Although critically important, such interventions address only some of the multitude of factors along all phases of the diagnostic process that can contribute to missed opportunities in cancer diagnosis. In addition, they tend to focus on primary care rather than keeping a system-wide focus and addressing factors during the medical encounter as well as the performance of tests and follow-up and coordination beyond the initial clinical encounter. Further, in both primary and secondary care we are yet to understand why physicians (both generalists and specialists) at times do not recognise or act upon obvious alarm symptoms or signs of cancer. Factors such as doctors' tiredness or stress, information overload (‘alert fatigue'), poor communication between primary and secondary care, and team dynamics are likely to be involved in such events (Singh *et al*, 2012a, 2013b). In contemporary complex and information technology-enabled health-care environments, acquiring such an understanding requires disciplinary inputs from the fields of psychology and behavioural/social sciences, human factors, systems engineering and clinical informatics. A systems approach to the problem of diagnostic safety is needed, as it is currently applied to surgical safety (Vincent *et al*, 2004). Interventions focussing only one factor—for example, solely aiming to optimise clinical reasoning during primary care encounters—might not be comprehensive enough, given that most instances of missed diagnostic opportunities typically involve several contributing factors.

We thus recommend that multi-disciplinary and multi-faceted approaches be developed to target the various phases in the diagnostic process where missed opportunities occur. These interventions should address the full range of contributing factors beyond mere individual cognitive or system capacity constraints. Because cancer diagnosis is distributed across time and place and involves interactions among multiple human and system components, these approaches should aim to strengthen both human and system performance and account for concepts such as shared mental models, distributed cognition and optimal technology use (Karsh *et al*, 2006; Henriksen and Brady, 2013; Smith *et al*, 2014). Newer forms of diagnostic and information technologies are being suggested to improve the diagnosis of cancer (e.g., electronic health records to enable informational continuity between different providers) requiring input from the field of clinical informatics (El-Kareh *et al*, 2013). However, although technological innovation can be helpful, many factors unrelated to technology can contribute to missed opportunities across all phases of the diagnostic process and must also be addressed. This can be achieved by a deeper understanding of ‘socio-technical' factors implicated in missed opportunities (workflow and organisational factors among others; Sittig and Singh, 2010). Multi-faceted approaches will enrich the spectrum of intervention targets, beyond facilitation of symptom recognition or access to specialist diagnostic assessment.

In conclusion, we call for more multi-disciplinary research that targets factors contributing to missed opportunities in all phases of the diagnostic process. The fields of organisational and cognitive psychology, human factors science and clinical informatics can all provide valuable insights into this emerging research agenda. Building on current approaches, we have provided a theoretical basis for the development of future interventions to shorten diagnostic intervals post presentation. The conceptual foundation we provide could motivate multi-disciplinary and multi-faceted strategies aimed at minimising the occurrence of missed diagnostic opportunities in cancer, enriching current approaches that principally focus on clinical decision support or on widening access to investigations.

## Figures and Tables

**Table 1:** Future initiatives and research agenda to address missed opportunities

**Possible practical steps**/**policies**	**Future research agenda**	**Potential fields involved**^a^
**Re-engineering of medical consultation norms and improving the quality of history taking and examination**		
Allocation of additional consultation time, particularly for infrequent attenders or multi-morbid patients (Andersen *et al*, 2014; McCartney, 2014) Patient engagement initiatives bolstering confidence in communicating symptoms and in not feeling that they ‘are wasting the doctors' time' (Andersen *et al*, 2011; Forbes *et al*, 2013) Ensuring formal or informal (e.g., family/relative) translation services for patients who are not competent in routine consultation language (e.g., ethnic minority patients)	Further quantification and qualification of mechanisms leading to ineffective symptom communication or elicitation during the patient encounter, and its implications	Social science Behavioural science Medical anthropology Policy Primary care Health services research
**Reducing cognitive or system barriers to optimal initial clinical assessment and reasoning**		
Effective decision support tools/diagnostic checklists to help minimise the risk of not thinking about a diagnosis of cancer in patients with common presentations and those with lower risk/atypical symptoms (Ely *et al*, 2011; Hamilton *et al*, 2013) Optimising decision making about investigation plans by increasing capacity/enabling prompt primary care access to specialist investigations and their prompt reporting Cultural shift towards accepting ‘low diagnostic hit rates'. Re-modelling of diagnostic quality norms from kudos given to doctors for ‘being right' to kudos given to doctors for ‘being safe'	Further evaluation of the use and impact of clinical decision support tools and diagnostic checklists in randomised controlled trials Evaluate the impact of additional consultation time, diagnostic services reorganisation, and increasing capacity on measures of diagnostic quality and safety	Clinical informatics Cognitive psychology Human factors Policy Primary care Health services research
**Improving diagnostic test performance, interpretation and follow-up**		
Development of resilient/fail-safe systems for following up and/or rebooking patient ‘no show' instances (for test performance or ‘expectant management' follow-up), or patients with negative test results but persistent/evolving symptoms Reorganisation of diagnostic pathways to allow for multi-processing of tests in ‘one-stop' clinics (all tests ‘under one roof, during one day') and wider access to specialist investigations Empowering of patients regarding outcomes of diagnostic investigations. Solutions may include sharing of patient records and enabling active chasing of test results by patients (Feeley and Shine, 2011) Effective electronic health record-enabled systems to ensure that diagnostic tests with abnormal results are acted upon by the relevant clinician Use of ‘navigators' (volunteers or professional staff) for complex cases (e.g., multiple tests, high/worsening symptom burden, patients with poor social support and/or other communication or transport difficulties; (Press, 2014)	Robustly evaluate the effectiveness of new models of diagnostic care (Guldbrandt *et al*, 2014) Explore optimal back-up tracking support systems for optimising diagnostic safety in electronic health record-enabled systems	Organisational psychology Human factors Clinical informatics Systems engineering Behavioural science Social science Policy Health services research Nursing

a This is an indicative list of relevant disciplines that should not be considered as ‘future proof': input from scientific fields beyond those mentioned may also be needed, as knowledge in this area is evolving.
